# Supplementary material for: Sororin is an evolutionary conserved antagonist of WAPL
Source: Nat Commun. 2024 Jun 3;15:4729. doi: 10.1038/s41467-024-49178-0 (PMC11148194; doi:10.1038/s41467-024-49178-0)
Supplement: Supplementary file 1 — Supplementary information [file 41467_2024_49178_MOESM1_ESM.pdf]

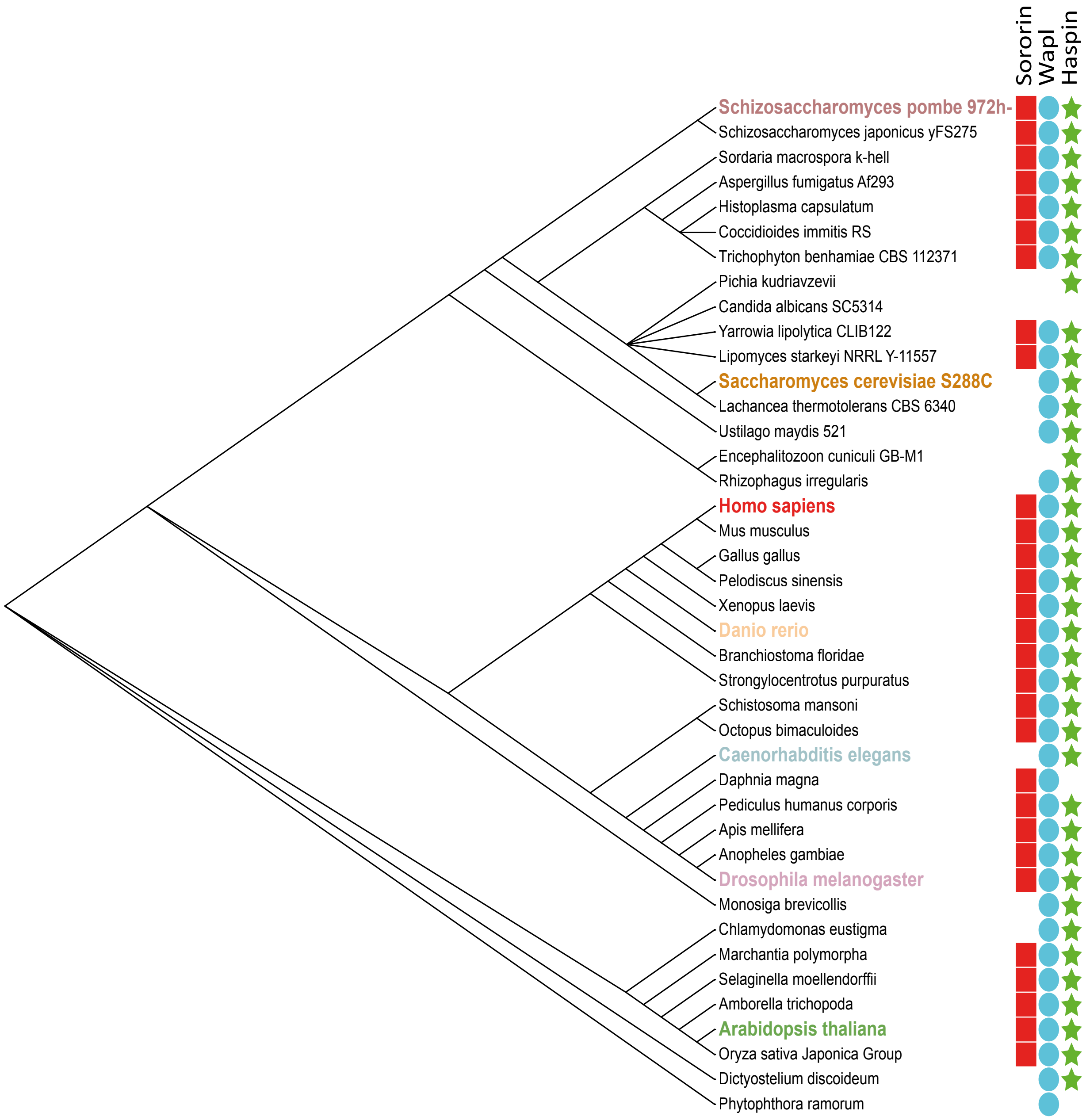

Supplementary Figure 1 (supporting Figure 1)**.**

Taxonomic tree of Sororin, Wapl, and Haspin containing species. Species were selected for a wide phylogenetic range and were mapped on a taxonomical hierarchy derived from NCBI Taxonomy Common Tree. The distribution of Sororin (red square), Wapl (light blue circle) or Haspin (green asterisk) proteins is indicated on the right. Sororin homologs are widespread in animal, fungi, and plant kingdoms, but are missing in slime molds, such as *Dictyostelium discoideum*, and in Oomycetes such as Phytophthora ramorum. We identified Sororin candidates in Saccharomycetes, such as *Yarrowia lipolytica*, but failed in species close to (and including) *Saccharomyces cerevisiae*, in which Wapl and Haspin relatives are present.

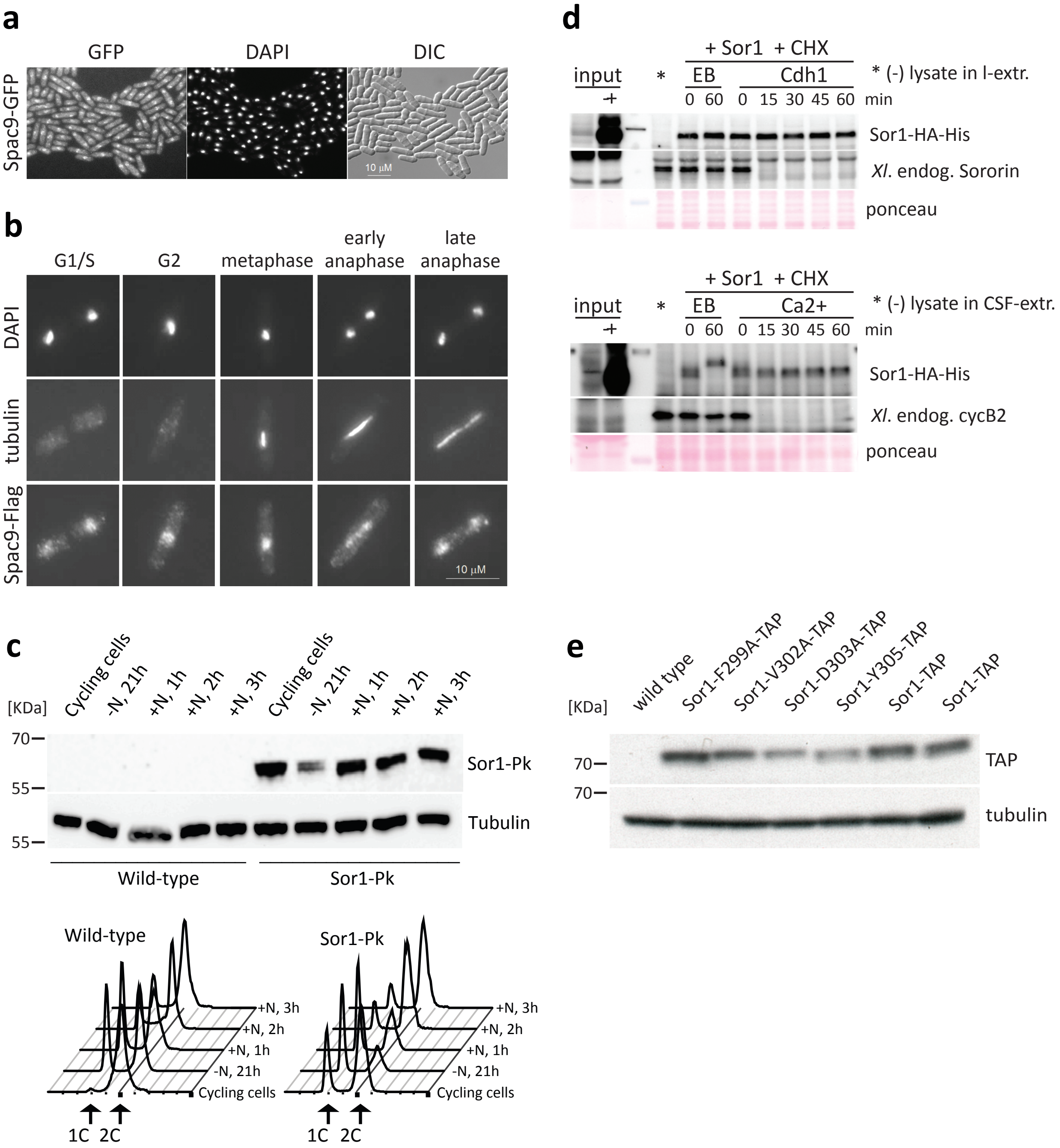

Supplementary Figure 2 (supporting Figure 2).

Subcellular localization, APC/C degradation assay and expression analysis of *S. pombe* Sor1. **a, b** Sor1 localizes to nucleus throughout the cell cycle. Cycling *S. pombe* cells expressing Sor1-GFP were fixed, stained with DAPI, and analyzed by fluorescence microscopy (**a**). Cycling *S. pombe* cells expressing Sor1-Flag were fixed and stained with antibodies against Flag and tubulin. Nuclei were visualized by DAPI staining (**b**). **c** Sor1 protein levels are lower in nitrogen starved/G1 cells. Wild-type and Sor1-Pk expressing cells were arrested in G1 by nitrogen starvation for 21 h at 30°C (-N, 21h). At t=0 cells were refed and harvested at 1 h (+N, 1h), 2 h (+N, 2h) and 3 h (+N, 3h). Western blot analysis (upper panel) shows Sor1-Pk levels over time with α-tubulin being used as loading control. To measure the DNA content, DNA was stained with Sytox Green and cells were analyzed by flow cytometry (lower panel). The experi-

ment was repeated twice with similar results. **d** *in vitro* assay shows no evidence that Sor1 is an APC/C substrate. In vitro-translated Sor1-HA-His was added to either interphase (upper panel) or meiotic metaphase-arrested CSF extract (lower panel) in the presence of cycloheximide (CHX). In upper panel, buffer (EB) or Cdh1 was then added to the extract and aliquoted for western blot analysis at indicated time points after EB or Cdh1 addition. In lower panel, EB or CaCl 2 was added and aliquoted for western blot analysis at indicated time points after EB or CaCl2 addition. Two independent experiments were performed and similar results were obtained. **e** Mutating conserved Sor1 residues only slightly reduces Sor1 protein levels. Proteins extracted from cycling cells were analyzed by gel electrophoresis and Western blotting using anti-tubulin antibodies. The TAP epitope was detected using PAP antibodies (rabbit anti-peroxidase antibody linked to peroxidase). The experiment was repeated twice with similar results.

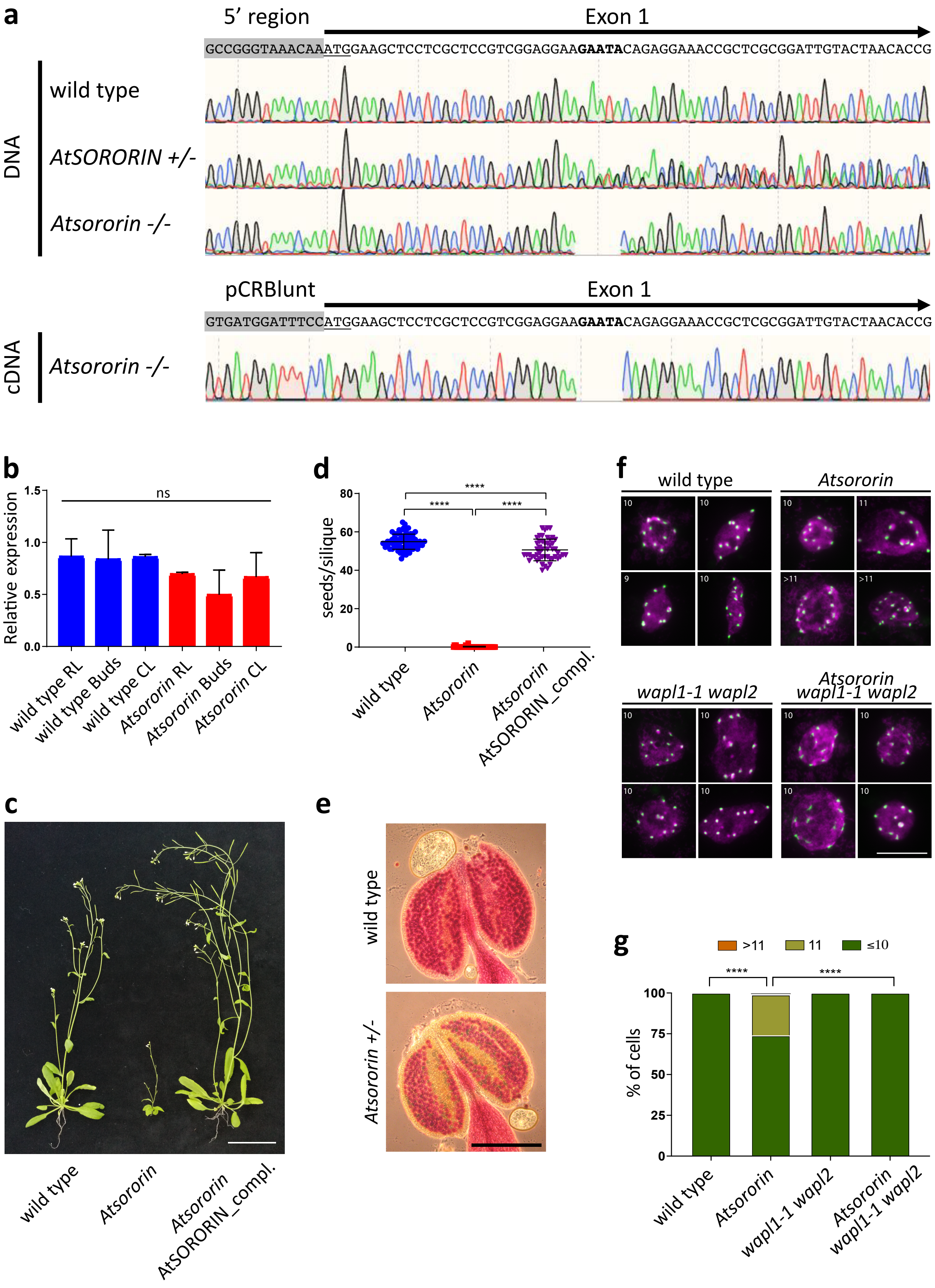

Supplementary Figure 3 (supporting Figures 3 and 4)**.**

In-depth characterization of the *Atsororin* mutant line: **a** Sequencing genomic DNA across the *AtSORORIN* gene displaying a wild-type sequence, a characteristic sequence from a heterozygous plant and a homozygous *Atsororin* mutant (upper panel). Sequencing the 5' region of the mRNA

(cDNA) isolated from wild-type (not shown) or homozygous *Atsororin* mutant plants (lower panel). **b** Analysis of the relative expression of the *AtSORORIN* gene in wild-type and homozygous *Atsororin* mutant in different tissues (RL – rosette leaves; buds; CL – cauline leaves). No significant differences could be observed (Kruskal-Wallis ANOVA with Dunn's correction).

Meiotic and somatic *Atsororin* mutant phenotypes can be complemented with the wild type *AtSORORIN* gene: **c** Overall plant architecture and fertility are wild type-like in the complemented transgenic plant line, but not in the *Atsororin* mutant. **d** Seed counts demonstrate nearly wild type-like fertility of the complemented transgenic plant line, but sterility in the *Atsororin* mutant. Unpaired Mann-Whitney test has been applied (****$p<0.0001$). **e** Alexander staining to evaluate pollen viability in wild-type (n = 8), homozygous (-/-) *Atsororin* mutant (n = 20) and heterozygous (+/–) *Atsororin* mutant (n = 3) plants. Red cytoplasm (Fuchsine red) with a thin green pollen wall (Malachite green) staining indicates viable pollen. Non-viable pollen grains show just green staining. Scale bar = 200 µm.

Somatic defects in *Atsororin* mutants are tissue-specific and WAPL-dependent. **f** Spreads of cell nuclei from rosette leaf cells. DNA was stained with DAPI (magenta) and fluorescence *in situ* hybridization (FISH) was performed to detect centromeric regions (green). Interphase stages were analyzed for wild type plants and *Atsororin*, *wapl1-1 wapl2* and *Atsororin wapl1-1 wapl2* mutants. The number of centromeric signals is indicated in the top left corner. Scale bar = 10µm. **g** Quantification of centromeric-FISH signals in interphase leaf nuclei. *Atsororin* mutants (n = 52) have a significantly higher number of cells that have more than 10 signals, when compared to wild type (n = 84), *wapl1-1 wapl2* (n = 68) and *Atsororin wapl1-1 wapl2* (n = 82). Fisher's exact test was performed (****$p<0.0001$).

The term "*Atsororin* mutant" refers to the homozygous mutant allele configuration if not explicitly stated otherwise.

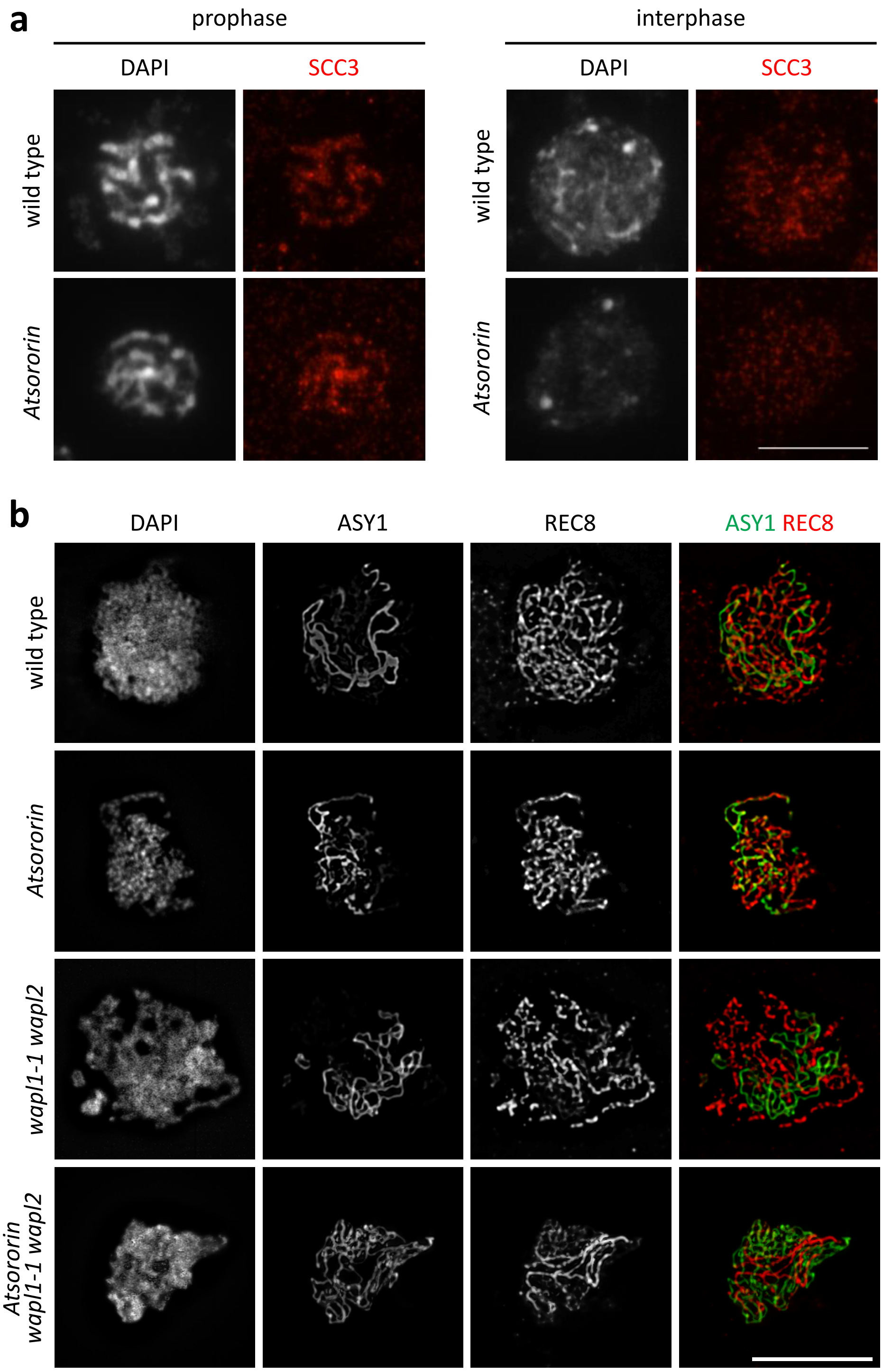

Supplementary Figure 4 (supporting Figure 7)**.**

a Immunolocalization of SCC3 in interphase and prophase nuclei of somatic cells obtained from inflorescences. No difference in localization and abundance could be observed when comparing cells from wild type and *Atsororin* mutants. Chromosomes were stained with DAPI. Scale bar = 10 µm. **b** Immunolocalization of the axis protein ASY1 and the meiosis-specific cohesin subunit REC8 in male meiocytes during late zygotene in wild type plants and *Atsororin*, *wapl1-1 wapl2* and *Atsororin wapl1-1 wapl2* mutants. Absence of AtSORORIN does not influence their time of deposition or their relative localisation on meiotic chromosomes. For each genotype at least 3 pictures all showing similar results were acquired. Scale bar = 10 µm.
