# Supplementary material for: Sororin is an evolutionary conserved antagonist of WAPL
Source: Nat Commun. 2024 Jun 3;15:4729. doi: 10.1038/s41467-024-49178-0 (PMC11148194; doi:10.1038/s41467-024-49178-0)
Supplement: Supplementary file 3 — Description of Additional Supplementary Files [file 41467_2024_49178_MOESM3_ESM.pdf]

Description of Additional Supplementary Files

File Name: Supplementary Movie 1
Description: Cell organization of the *A. thaliana sororin* mutant root tip.

File Name: Supplementary Movie 2
Description: Cell organization of the *A. thaliana wapl1 wapl2 sororin* triple mutant root tip.

File Name: Supplementary Movie 3
Description: Cell organization of the *A. thaliana wapl1 wapl2* double mutant root tip

File Name: Supplementary Movie 4
Description: Cell organization of the *A. thaliana* Col-0 wild type root tip.
