# Supplementary material for: Sororin is an evolutionary conserved antagonist of WAPL
Source: Nat Commun. 2024 Jun 3;15:4729. doi: 10.1038/s41467-024-49178-0 (PMC11148194; doi:10.1038/s41467-024-49178-0)
Supplement: Supplementary file 8 — Reporting Summary [file 41467_2024_49178_MOESM8_ESM.pdf]

nature portfolio

Corresponding author(s): Gregan J., Peters J.-M., Schlögelhofer P.

Last updated by author(s): 2024 03 15

Reporting Summary

Nature Portfolio wishes to improve the reproducibility of the work that we publish. This form provides structure for consistency and transparency in reporting. For further information on Nature Portfolio policies, see our Editorial Policies and the Editorial Policy Checklist.

Statistics

For all statistical analyses, confirm that the following items are present in the figure legend, table legend, main text, or Methods section.

n/a	Confirmed	
☐	☒	The exact sample size (*n*) for each experimental group/condition, given as a discrete number and unit of measurement
☐	☒	A statement on whether measurements were taken from distinct samples or whether the same sample was measured repeatedly
☐	☒	The statistical test(s) used AND whether they are one- or two-sided<br>*Only common tests should be described solely by name; describe more complex techniques in the Methods section.*
☐	☒	A description of all covariates tested
☐	☒	A description of any assumptions or corrections, such as tests of normality and adjustment for multiple comparisons
☐	☒	A full description of the statistical parameters including central tendency (e.g. means) or other basic estimates (e.g. regression coefficient) AND variation (e.g. standard deviation) or associated estimates of uncertainty (e.g. confidence intervals)
☐	☒	For null hypothesis testing, the test statistic (e.g. *F*, *t*, *r*) with confidence intervals, effect sizes, degrees of freedom and *P* value noted<br>*Give P values as exact values whenever suitable.*
☒	☐	For Bayesian analysis, information on the choice of priors and Markov chain Monte Carlo settings
☒	☐	For hierarchical and complex designs, identification of the appropriate level for tests and full reporting of outcomes
☒	☐	Estimates of effect sizes (e.g. Cohen's *d*, Pearson's *r*), indicating how they were calculated

Our web collection on statistics for biologists contains articles on many of the points above.

Software and code

Policy information about availability of computer code

Data collection	*Provide a description of all commercial, open source and custom code used to collect the data in this study, specifying the version used OR state that no software was used.*
Data analysis	*Provide a description of all commercial, open source and custom code used to analyse the data in this study, specifying the version used OR state that no software was used.*

For manuscripts utilizing custom algorithms or software that are central to the research but not yet described in published literature, software must be made available to editors and reviewers. We strongly encourage code deposition in a community repository (e.g. GitHub). See the Nature Portfolio guidelines for submitting code & software for further information.

Data

Policy information about availability of data

All manuscripts must include a data availability statement. This statement should provide the following information, where applicable:

- Accession codes, unique identifiers, or web links for publicly available datasets
- A description of any restrictions on data availability
- For clinical datasets or third party data, please ensure that the statement adheres to our policy

All data supporting the findings of this study are available within the paper and its Supplementary Information. Additionally a source data file has been created and submitted containing uncropped blots and gels, all quantitative data and the corresponding statistical analyses.

Research involving human participants, their data, or biological material

Policy information about studies with human participants or human data. See also policy information about sex, gender (identity/presentation), and sexual orientation and race, ethnicity and racism.

Reporting on sex and gender	*Use the terms sex (biological attribute) and gender (shaped by social and cultural circumstances) carefully in order to avoid confusing both terms. Indicate if findings apply to only one sex or gender; describe whether sex and gender were considered in study design; whether sex and/or gender was determined based on self-reporting or assigned and methods used. Provide in the source data disaggregated sex and gender data, where this information has been collected, and if consent has been obtained for sharing of individual-level data; provide overall numbers in this Reporting Summary. Please state if this information has not been collected. Report sex- and gender-based analyses where performed, justify reasons for lack of sex- and gender-based analysis.*
Reporting on race, ethnicity, or other socially relevant groupings	*Please specify the socially constructed or socially relevant categorization variable(s) used in your manuscript and explain why they were used. Please note that such variables should not be used as proxies for other socially constructed/relevant variables (for example, race or ethnicity should not be used as a proxy for socioeconomic status). Provide clear definitions of the relevant terms used, how they were provided (by the participants/respondents, the researchers, or third parties), and the method(s) used to classify people into the different categories (e.g. self-report, census or administrative data, social media data, etc.) Please provide details about how you controlled for confounding variables in your analyses.*
Population characteristics	*Describe the covariate-relevant population characteristics of the human research participants (e.g. age, genotypic information, past and current diagnosis and treatment categories). If you filled out the behavioural & social sciences study design questions and have nothing to add here, write "See above."*
Recruitment	*Describe how participants were recruited. Outline any potential self-selection bias or other biases that may be present and how these are likely to impact results.*
Ethics oversight	*Identify the organization(s) that approved the study protocol.*

Note that full information on the approval of the study protocol must also be provided in the manuscript.

Field-specific reporting

Please select the one below that is the best fit for your research. If you are not sure, read the appropriate sections before making your selection.

- [x] Life sciences
- [ ] Behavioural & social sciences
- [ ] Ecological, evolutionary & environmental sciences

For a reference copy of the document with all sections, see nature.com/documents/nr-reporting-summary-flat.pdf

Life sciences study design

All studies must disclose on these points even when the disclosure is negative.

Sample size	The severe phenotype of the Atsororin mutant plant and its distorted transmission compromised the number of surviving individuals and therefore limited sample collection. As the observed differences were severe, the relatively small number of individual plants/cells observed showed significant differences for the tested features.
Data exclusions	No data was excluded.
Replication	All experiments either state the replication numbers or the numbers of used individuals.
Randomization	All plants were grown side-by-side at the same time and random plants of the needed genotype chosen for analysis. Yeast cultures were also grown side-by-side and experiments repeated.
Blinding	Mutant phenotypes were too strong and obvious. Blinding would not make any sense.

Reporting for specific materials, systems and methods

We require information from authors about some types of materials, experimental systems and methods used in many studies. Here, indicate whether each material, system or method listed is relevant to your study. If you are not sure if a list item applies to your research, read the appropriate section before selecting a response.

Materials & experimental systems

n/a	Involved in the study
☐	☒ Antibodies
☒	☐ Eukaryotic cell lines
☒	☐ Palaeontology and archaeology
☒	☐ Animals and other organisms
☒	☐ Clinical data
☒	☐ Dual use research of concern
☐	☒ Plants

Methods

n/a	Involved in the study
☒	☐ ChIP-seq
☒	☐ Flow cytometry
☒	☐ MRI-based neuroimaging

Antibodies

Antibodies used

Commercial antibodies used: Rabbit antiperoxidase antibody linked to peroxidase (PAP, Dako; 1:10000 dilution). Tubulin was detected using mouse-anti-α-tubulin antibody (Sigma-Aldrich T5168; 1:10000 dilution) and rabbit anti-mouse IgG-HRP secondary antibody (Santa Cruz Biotechnology; 1:5000 dilution). GFP-tagged proteins were detected using mouse anti-GFP antibody (Roche 1814460, 1:1000 dilution) and anti-mouse-HRP antibody (Amersham, 1:5000). Pk-tagged proteins were detected using mouse-anti-Pk (V5) antibody (Serotec; 1:2000 dilution) and goat anti-mouse IgG-HRP secondary antibody (Santa Cruz Biotechnology; 1:5000 dilution) in 0.1% PBS-T. Myc-tagged proteins were detected using rabbit c-Myc antiserum (CM-100, Gramsch, Germany, 1:10000 dilution) and secondary mouse anti-rabbit-IgG antibody conjugated to HRP (sc-2357, Santa Cruz Biotechnology, 1:20000 dilution). Anti-Pk antibody (mouse-anti-Pk (V5) antibody, Invitrogen R960-25, 1:2000 dilution) and goat anti-mouse IgG-HRP secondary antibody (Jackson ImmunoResearch 115-035-033, 1:10000 dilution) were used to detect Sor1-Pk. Anti-tubulin antibody (Sigma-Aldrich T5168; 1:10000 dilution) and rabbit anti-mouse IgG-HRP secondary antibody (Jackson ImmunoResearch 115-035-033, 1:10000 dilution) were used to detect alpha tubulin as a loading control. The following secondary antibodies are all commercially available and were used as follows: anti-guinea pig conjugated to Alexa Fluor 488 (1:400)(Invitrogen; 10123952), anti-rabbit conjugated to Alexa Fluor 568 (1:400) (Invitrogen; 10032302) and anti-rat conjugated to Alexa Fluor 647 (1:200)(Invitrogen; 10748034).
Primary antibodies produced in the Schlögelhofer Lab or obtained from collaborators: 1:10000 anti-ASY1 raised in guinea pig (Reference 105 in manuscript and see below), 1:500 anti-ZYP1 raised in rat (Reference 107), 1:500 anti-SCC3 raised in rabbit (Refernece 108) and 1:250 anti-REC8 raised in rabbit (Reference 109).

Validation

Validation of commercial primary and secondary antibodies according to company specifications.
Validation of antibodies raised in the Schlögelhofer Lab or by collaborators:
105. Sims, J., Copenhaver, G. P. & Schlögelhofer, P. Meiotic DNA repair in the nucleolus employs a nonhomologous end-joining mechanism. Plant Cell 31, 2259–2275 (2019).
107. Higgins, J. D., Sanchez-Moran, E., Armstrong, S. J., Jones, G. H. & Franklin, F. C. H. The Arabidopsis synaptonemal complex protein ZYP1 is required for chromosome synapsis and normal fidelity of crossing over. Genes Dev. 19, 2488–2500 (2005).
108. Chelysheva, L. et al. AtREC8 and AtSCC3 are essential to the monopolar orientation of the kinetochores during meiosis. J. Cell Sci. 118, 4621–4632 (2005).
109. Cromer, L. et al. Centromeric cohesion is protected twice at meiosis, by SHUGOSHINs at anaphase i and by PATRONUS at interkinesis. Curr. Biol. 23, 2090–2099 (2013).
109

Plants

Seed stocks

The Arabidopsis thaliana Columbia (Col-0) ecotype was used as wild-type reference. Atsororin mutant plants were generated via CRISPR-Cas9 (see below). The wapl1-1 wapl2 double mutant (SALK_108385, SALK_127445) 35 was crossed with heterozygous AtSORORIN +/- mutant to obtain the Atsororin wapl1-1 wapl2 triple mutant.
The Atsororin mutant was generated by using the CRISPR-CAS9 technology. The gRNA sequence 5’-CCGTCGGAGGAAGAATACAG-3’ is specific to exon 1 of the ATSORORIN gene (At3g56250) and induces cleavage a few nucleotides downstream of the ATG codon. The gRNA was cloned into pGGE000-EF_pChimera2, and together with the Cas9 promoter in pGGA000-AB_PcUbi, the Cas9 version in pGGB000-BC_PuCas9 and the Cas9 terminator in pGGC000-CD_PeaTer further subcloned into the destination vector pGGZ003 utilizing the GOLDENGATE technique. The final plasmid was used to transform Col-0 plants by using the floral dip method 100. Transgenic plants grown on soil were identified and selected by their resistance to the herbicide Basta (applied by spraying 13.5 mg/l). For subsequent generations we screened for the absence/presence of the BASTA resistance gene (PAT) using the primers 35Sp_Fwd and Basta_Rev. Offspring of the initial transformants with or without the transgene were analysed for the presence of a mutation in the first exon 1 of the AtSORORIN gene. To do so, PCR amplicons were generated using the primers Sororin_geno_Fwd and Sororin_geno_Rev and subsequently sequenced with the primer Sororin_sequencing (Table 2). Plants with a mutation signature were grown for one or two more generations to identify individuals that inherited the mutation. We finally obtained a line without transgene and a stable heterozygous mutation in the AtSORORIN gene (Figure 1). The Atsororin mutant line contains a 5bps deletion within the first exon, 25 nucleotides down-stream of the ATG start codon (Figure 3a; Supplementary Figure 3a). It results in a premature TAA stop codon after generating a short peptide of 18 amino acid residues.

Novel plant genotypes

Describe the methods by which all novel plant genotypes were produced. This includes those generated by transgenic approaches, gene editing, chemical/radiation-based mutagenesis and hybridization. For transgenic lines, describe the transformation method, the number of independent lines analyzed and the generation upon which experiments were performed. For gene-edited lines, describe the editor used, the endogenous sequence targeted for editing, the targeting guide RNA sequence (if applicable) and how the editor was applied.

Authentication

Describe any authentication procedures for each seed stock used or novel genotype generated. Describe any experiments used to assess the effect of a mutation and, where applicable, how potential secondary effects (e.g. second site T-DNA insertions, mosiacism, off-target gene editing) were examined.
