# Supplementary material for: Sororin is an evolutionary conserved antagonist of WAPL
Source: Nat Commun. 2024 Jun 3;15:4729. doi: 10.1038/s41467-024-49178-0 (PMC11148194; doi:10.1038/s41467-024-49178-0)
Supplement: Supplementary file 9 — Source Data [file 41467_2024_49178_MOESM9_ESM.zip › Prusen Mota et al_Source_Data (1).pdf]

statistics

FIGURE 2a **Split sister centromeres**

All samples follow gaussian distribution (D'Agostino & Pearson normality test)
Unpaired t-test

	n	mean	sd
wild type	45	41	1,4
sor1	39	49	3,5
wpl1 -mutant	37	40	1,7
sor1 -mutant *wpl1* -mutant	50	42	1,9

p-value		
	sor1- mutant	*sor1* -mutant *wpl1* -mutant
wild type	0.002 **	<0.367 ns

FIGURE 2b **Missegregation**

All samples follow gaussian distribution (D'Agostino & Pearson normality test)
Unpaired t-test

	n	mean	sd
wild type	4	0	
sor1 -mutant	4	0	
eso1-ts	4	2	0,7
eso1-ts sor1 -mutant	4	6	1,4
mis4-ts	4	0	
mis4-ts sor1-mutant	4	2	0,7

p-value		
	eso1-ts sor1 -mutant	*mis4-ts sor1-mutant*
eso1-ts	0.005 **	
mis4-ts		0.011 *

FIGURE 2b **Lagging chromosomes**

All samples follow gaussian distribution (D'Agostino & Pearson normality test)
Unpaired t-test

	n	mean	sd
wild type	4	1	0,7
sor1 -mutant	4	1	0,7
eso1-ts	4	20	2,5
eso1-ts sor1 -mutant	4	33	4,4
mis4-ts	4	2	0,7
mis4-ts sor1-mutant	4	4	1

p-value		
	eso1-ts sor1 -mutant	*mis4-ts sor1-mutant*
eso1-ts	0.004 **	
mis4-ts		0.02 *

FIGURE 3d Root lenght

One of the samples does not follow gaussian distribution (D'Agostino & Pearson normality test)
Unpaired Mann-Whitney test

	n	mean	sd	gaussian dist.?
Wild type	139	4,447	0,8771	no
sororin	23	0,5696	0,339	yes
wapl1-1 wapl2	113	4,2	0,9895	yes
sororin wapl1-1 wapl2	113	4,005	1,373	yes

p-value			
	sororin	*wapl1-1 wapl2*	*sororin wapl1-1 wapl2*
Wild type	<0.000000000000001 ****	0,029409052572133 *	0,005110866194875 **
sororin			
wapl1-1 wapl2	<0.000000000000001 ****		
sororin wapl1-1 wapl2	<0.000000000000001 ****	0,286588677752765 ns	

FIGURE 3e Seeds/silique

One of the samples does not follow gaussian distribution (D'Agostino & Pearson normality test)
Unpaired Mann-Whitney test

	n	mean	sd	gaussian dist.?
Wild type	74	54,92	3,93	yes
sororin	52	0,096	0,3575	no
wapl1-1 wapl2	144	36,2	9,301	no
sororin wapl1-1 wapl2	166	5,373	3,56	no

p-value			
	sororin	*wapl1-1 wapl2*	*sororin wapl1-1 wapl2*
Wild type	<0.000000000000001 ****	<0.000000000000001 ****	<0.000000000000001 ****
sororin			
wapl1-1 wapl2	<0.000000000000001 ****		
sororin wapl1-1 wapl2	<0.000000000000001 ****	<0.000000000000001 ****	

FIGURE 3f Segregation self-pollinated AtSORORIN +/-

Contingency table with two classes ->	Observed vs. Expected
Chi-square	

	n	% observed	% expected
+/+	160	47	25
+/-	168	50	50
-/-	11	3	25

P value	<0.0000000001
significance	****

FIGURE 3g — Segregation reciprocal crosses

Contingency table with two classes ->	Observed vs. Expected
Two-sided Fischer´s exact test	

FEMALE	X	MALE
AtSORORIN +/-	X	Wild type

	n	% observed	% expected
+/+	88	54	50
+/-	75	46	50

P value	0,506
significance	ns

FEMALE	X	MALE
Wild type	X	*AtSORORIN +/-*

	n	% observed	% expected
+/+	87	91,5	50
+/-	8	8,5	50

P value	1.25 x10-10
significance	****

FIGURE 4b — Root interphase nuclei (CEN signals)

Contingency table with three classes ->	<=10, 11 or >11
Fisher's exact test	

	<=10	11	>11	n	mean	sd
Wild type	91	2	0	93	10,02	0,1458
sororin	4	1	29	34	16,82	3,688
wapl1-1 wapl2	69	1	3	73	10,32	1,657
sororin wapl1-1 wapl2	48	9	2	59	10,49	1,832

p-value

	sororin	*wapl1-1 wapl2*	*sororin wapl1-1 wapl2*
Wild type	<0.000000000000001 ****	0,188473202319178 ns	0,000739269058589 ***
sororin			
wapl1-1 wapl2	<0.000000000000001 ****		
sororin wapl1-1 wapl2	<0.000000000000001 ****	0,007276883442059 **	

FIGURE 4d — Inflorescence interphase nuclei (CEN signals)

Contingency table with three classes ->	<=10, 11 or >11
Fisher's exact test	

	<=10	11	>11	n	mean	sd
Wild type	224	0	0	224	10	0
sororin	233	29	4	266	10,26	1,246
wapl1-1 wapl2	236	2	0	238	10,01	0,09148
sororin wapl1-1 wapl2	233	1	2	236	10,09	0,9205

p-value

	sororin	*wapl1-1 wapl2*	*sororin wapl1-1 wapl2*
Wild type	0,000000002467865 ****	0,499375534082696 ns	0,499251681349105 ns
sororin			
wapl1-1 wapl2	0,000000146260013 ****		
sororin wapl1-1 wapl2	0,000000135696545 ****	0,434332960773726 ns	

FIGURE 4e **CEN distance prophase (inflorescence)**

All samples follow gaussian distribution (D'Agostino & Pearson normality test)
Unpaired t-test

	n	mean	sd	gaussian dist.?
Wild type	45	378,9	83,01	yes
sororin	39	457,6	112,5	yes
wapl1-1 wapl2	37	294,2	57,5	yes
sororin wapl1-1 wapl2	50	349,2	56,88	yes

p-value			
	sororin	*wapl1-1 wapl2*	*sororin wapl1-1 wapl2*
Wild type	0,000416986602970 ***	0,000001215578947 ****	0,042732258592203 *
sororin			
wapl1-1 wapl2	0,000000000019368 ****		
sororin wapl1-1 wapl2	0,000000061884407 ****	0,000027678991770 ****	

FIGURE 4f **CEN distance prometaphase (inflorescence)**

All samples follow gaussian distribution (D'Agostino & Pearson normality test)
Unpaired t-test

	n	mean	sd	gaussian dist.?
Wild type	42	562,9	91,71	yes
sororin	55	699	123,9	yes
wapl1-1 wapl2	43	453,9	85,24	yes
sororin wapl1-1 wapl2	49	446,1	78,04	yes

p-value			
	sororin	*wapl1-1 wapl2*	*sororin wapl1-1 wapl2*
Wild type	0,000000039111907 ****	0,000000195572818 ****	0,000000003387440 ****
sororin			
wapl1-1 wapl2	<0.000000000000001 ****		
sororin wapl1-1 wapl2	<0.000000000000001 ****	0,648524644385661 ns	

FIGURE 6c **MetaphaseI - ProphaseII**

Contingency table with three classes -> 10, 11-19 or 20
Fisher's exact test

	number of signals			
	10 FISH	11-19 FISH	20 FISH	n
Wild type	71	5	0	76
sororin	0	16	8	24
wapl1-1 wapl2	21	2	0	23
sororin wapl1-1 wapl2	2	27	2	31

p-value			
	sororin	*wapl1-1 wapl2*	*sororin wapl1-1 wapl2*
Wild type	<0.000000000000001 ****	0.6622 ns	<0.000000000000001 ****
sororin			
wapl1-1 wapl2	0,000000000023630 ****		
sororin wapl1-1 wapl2	0,017269065267743 *	0,000000000156288 ****	

	% of signals		
	10 FISH	11-19 FISH	20 FISH
Wild type	93,4	6,58	0
sororin	0	66,66	33,33
wapl1-1 wapl2	91,3	8,7	0
sororin wapl1-1 wapl2	6,45	87,1	6,45

FIGURE 6d **MetaphaseII**

Contingency table with three classes ->	20 paired, intermediate or 20 single
Fisher's exact test	

	number of signals			
	20 paired	int.	20 single	n
Wild type	17	0	0	17
sororin	0	7	6	13
wapl1-1 wapl2	10	0	0	10
sororin wapl1-1 wapl2	0	13	4	17

	% of signals		
	20 paired	int.	20 single
Wild type	100	0	0
sororin	0	54	46
wapl1-1 wapl2	100	0	0
sororin wapl1-1 wapl2	0	76	24

	p-value		
	sororin	*wapl1-1 wapl2*	*sororin wapl1-1 wapl2*
Wild type	0,000000008350044 ****	>0.999999999999999 ns	0,000000000857043 ****
sororin			
wapl1-1 wapl2	0,000000874075447 ****		
sororin wapl1-1 wapl2	0,255286642393086 ns	0,000000118535588 ****	

FIGURE 6e **Tetrad**

Contingency table with two classes ->	balanced or unbalanced
Two-sided Fisher´s exact test	

	number of signals		
	balanc.	unbalanc.	n
Wild type	33	0	33
sororin	0	25	25
wapl1-1 wapl2	21	7	28
sororin wapl1-1 wapl2	0	18	18

	% of signals	
	balanc.	unbalanc.
Wild type	100	0
sororin	0	100
wapl1-1 wapl2	75	25
sororin wapl1-1 wapl2	0	100

	p-value		
	sororin	*wapl1-1 wapl2*	*sororin wapl1-1 wapl2*
WT	<0.000000000000001 ****	0.0027 **	0,000000000000036 ****
sororin			
wapl1-1 wapl2	0,000000003763337 ****		
sororin wapl1-1 wapl2	>0.9999 ns	0,000000224074320 ****	

SUPPL. FIGURE 3b **qPCR of *AtSORORIN***

Legend

r- rosette leaves
b- buds
c- cauline leaves

1,2,3: different biological replicates

Kruskal-Wallis ANOVA with Dunn’s correction

Dunn's multiple comparisons test	Mean rank di	Significant?	Summary	Adjusted P Value	
wild type RL vs. wild type Buds	0	No	ns	>0.9999	A-B
wild type RL vs. wild type CL	0,6667	No	ns	>0.9999	A-C
wild type RL vs. *Atsororin* RL	4,667	No	ns	>0.9999	A-D
wild type RL vs. *Atsororin* Buds	8,333	No	ns	0,8309	A-E
wild type RL vs. *Atsororin* CL	5,333	No	ns	>0.9999	A-F
wild type Buds vs. wild type CL	0,6667	No	ns	>0.9999	B-C
wild type Buds vs. *Atsororin* RL	4,667	No	ns	>0.9999	B-D
wild type Buds vs. *Atsororin* Buds	8,333	No	ns	0,8309	B-E
wild type Buds vs. *Atsororin* CL	5,333	No	ns	>0.9999	B-F
wild type CL vs. *Atsororin* RL	4	No	ns	>0.9999	C-D
wild type CL vs. *Atsororin* Buds	7,667	No	ns	>0.9999	C-E
wild type CL vs. *Atsororin* CL	4,667	No	ns	>0.9999	C-F
Atsororin RL vs. *Atsororin* Buds	3,667	No	ns	>0.9999	D-E
Atsororin RL vs. *Atsororin* CL	0,6667	No	ns	>0.9999	D-F
Atsororin Buds vs. *Atsororin* CL	-3	No	ns	>0.9999	E-F

SOR qPCR		ACT7 qPCR	
Name	Ct SYBR	Name	Ct SYBR
wt r1	27,40	wt r1	20,70
wt r1	27,63	wt r1	20,64
wt r1	27,18	wt r1	20,57
wt r2	27,80	wt r2	20,77
wt r2	27,71	wt r2	21,03
wt r2	27,88	wt r2	20,77
wt r3	30,02	wt r3	21,81
wt r3	29,67	wt r3	22,03
wt r3	30,37	wt r3	21,96
wt b1	26,13	wt b1	19,48
wt b1	26,13	wt b1	19,45
wt b1	26,09	wt b1	19,47
wt b2	27,76	wt b2	19,81
wt b2	27,84	wt b2	19,95
wt b2	27,70	wt b2	19,84
wt b3	26,98	wt b3	19,33
wt b3	26,76	wt b3	19,39
wt b3	26,16	wt b3	19,33
wt c1	27,78	wt c1	21,75
wt c1	28,79	wt c1	21,74
wt c1	28,39	wt c1	21,61
wt c2	28,12	wt c2	21,11
wt c2	28,54	wt c2	21,26
wt c2	29,15	wt c2	21,29
wt c3	29,66	wt c3	22,02
wt c3	30,01	wt c3	22,00
wt c3	29,61	wt c3	21,94
sor r1	28,74	sor r1	21,70
sor r1	29,10	sor r1	21,99
sor r1	29,35	sor r1	21,88
sor r2	29,71	sor r2	22,36
sor r2	30,11	sor r2	22,45
sor r2	29,84	sor r2	22,46
sor r3	29,15	sor r3	20,92
sor r3	29,04	sor r3	21,18
sor r3	28,89	sor r3	20,96
sor b1	27,22	sor b1	19,41
sor b1	27,54	sor b1	19,54
sor b1	27,68	sor b1	19,56
sor b2	28,88	sor b2	20,30
sor b2	28,97	sor b2	20,34
sor b2	28,90	sor b2	20,44
sor b3	28,08	sor b3	20,16
sor b3	28,02	sor b3	20,21
sor b3	28,17	sor b3	20,21
sor c1	32,35	sor c1	24,62
sor c1	31,68	sor c1	24,62
sor c1	31,11	sor c1	24,59
sor c2	30,81	sor c2	24,41
sor c2	30,58	sor c2	24,36
sor c2	30,97	sor c2	24,15
sor c3	31,83	sor c3	23,57
sor c3	31,34	sor c3	23,59
sor c3	31,70	sor c3	23,76
H_2O	-	H_2O	35,97
H_2O	-	H_2O	36,39
H_2O	-	H_2O	34,83

SUPPL. FIGURE 3b **Seeds/silique**

One of the samples does not follow gaussian distribution (D'Agostino & Pearson normality test)
Unpaired Mann-Whitney test

	n	mean	sd	gaussian dist.?
Wild type	74	54,92	3,93	yes
sororin	52	0,096	0,3575	no
Complementation	50	50,6	5,64	yes

p-value		
	sororin	Complementation
Wild type	<0.000000000000001 ****	0,000007207460567 ****
sororin		
Complementation	<0.000000000000001 ****	

SUPPL. FIGURE 3g **Leaves interphase nuclei (CEN signals)**

Contingency table with three classes ->	<=10, 11 or >11
Fisher's exact test	

	<=10	11	>11	n	mean	sd
Wild type	84	0	0	84	10	0
sororin	39	13	1	53	10,3	0,5746
wapl1-1 wapl2	68	0	0	68	10	0
sororin wapl1-1 wapl2	82	0	0	82	10	0

p-value			
	sororin	*wapl1-1 wapl2*	*sororin wapl1-1 wapl2*
WT	0,000000507803019 ****		
sororin			
wapl1-1 wapl2	0,000003175664935 ****		
sororin wapl1-1 wapl2	0,000000630506854 ****		

Figure 2c

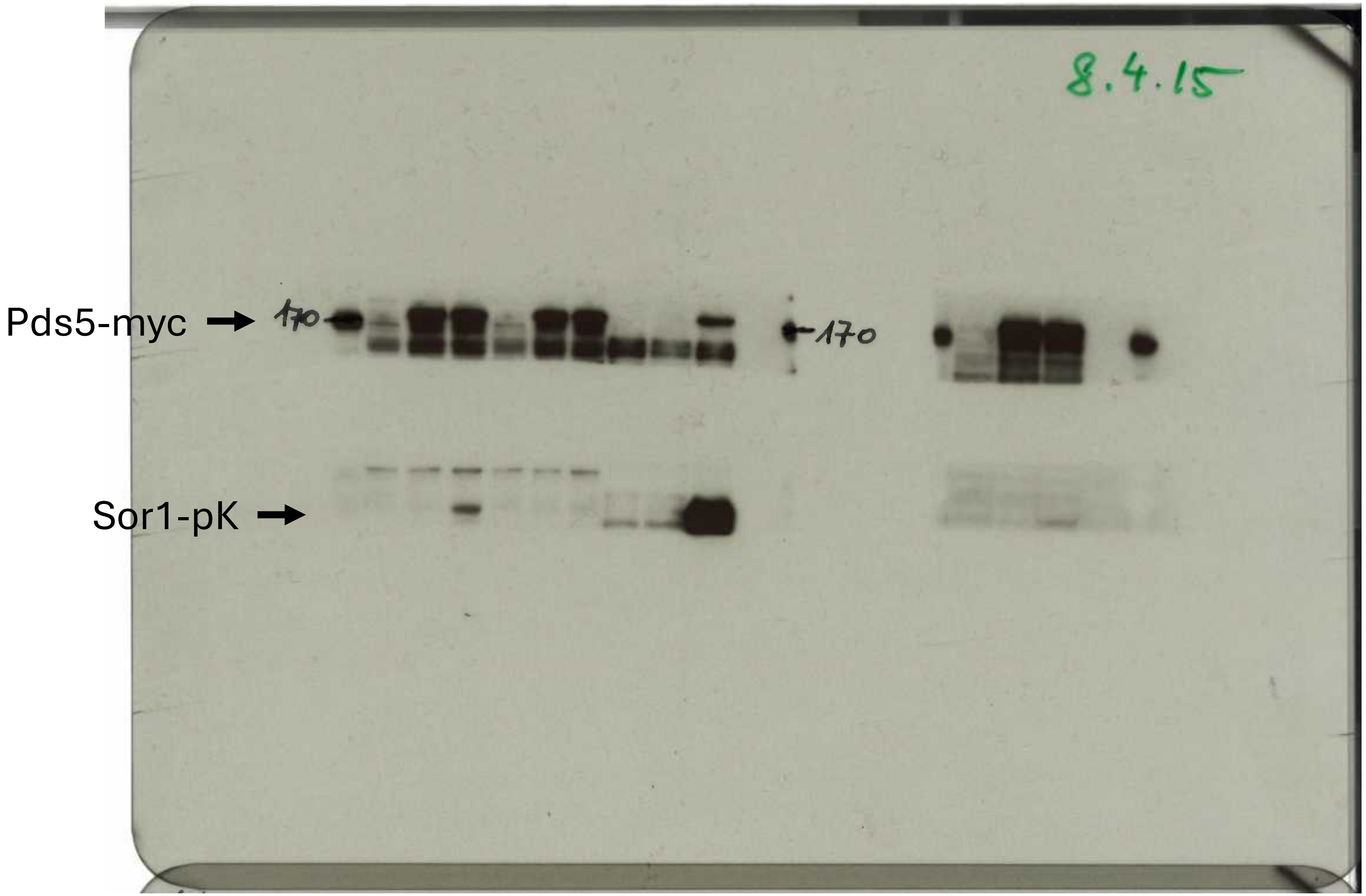

Figure 2d

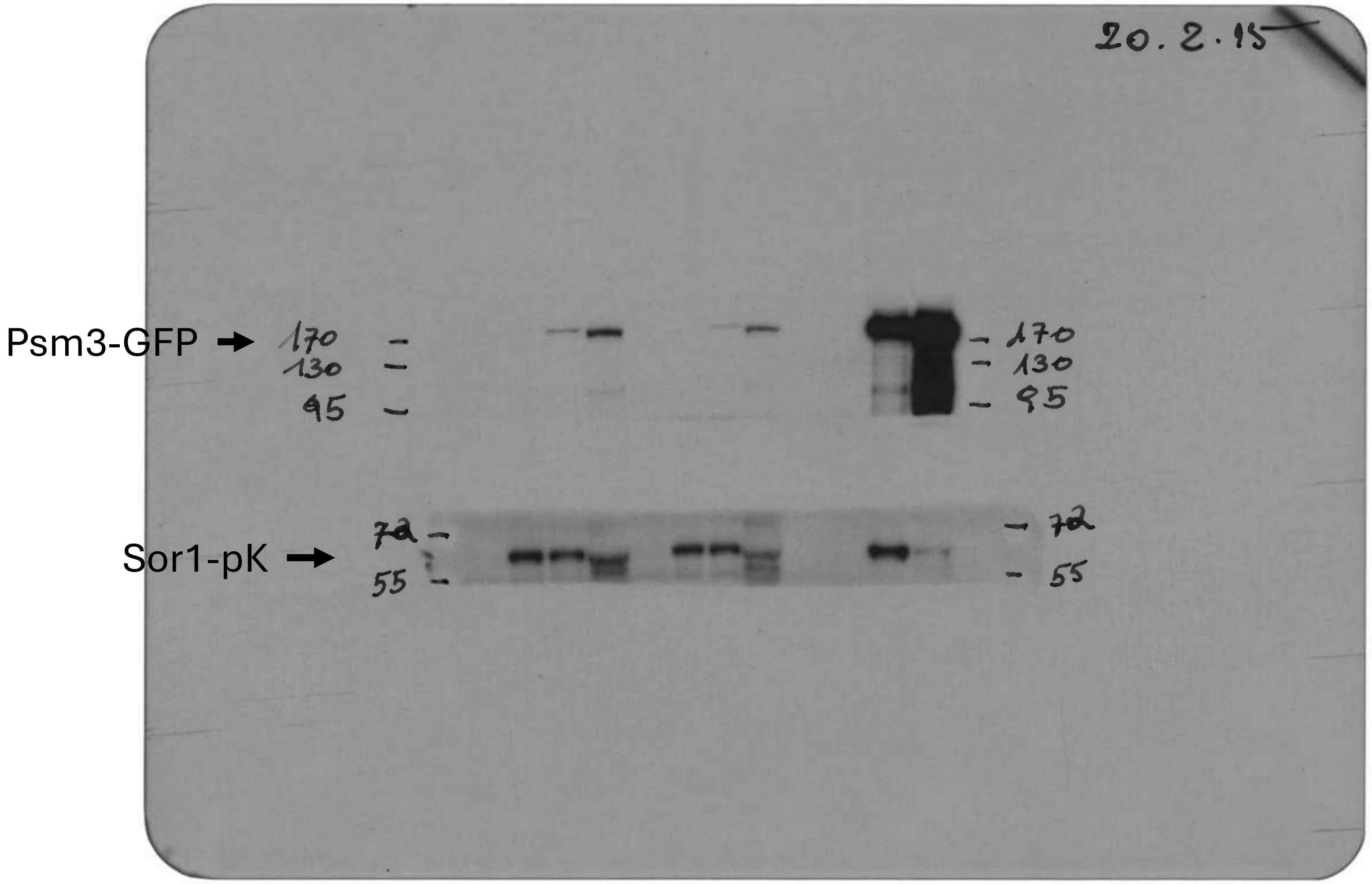

Supplementary Figure 2c

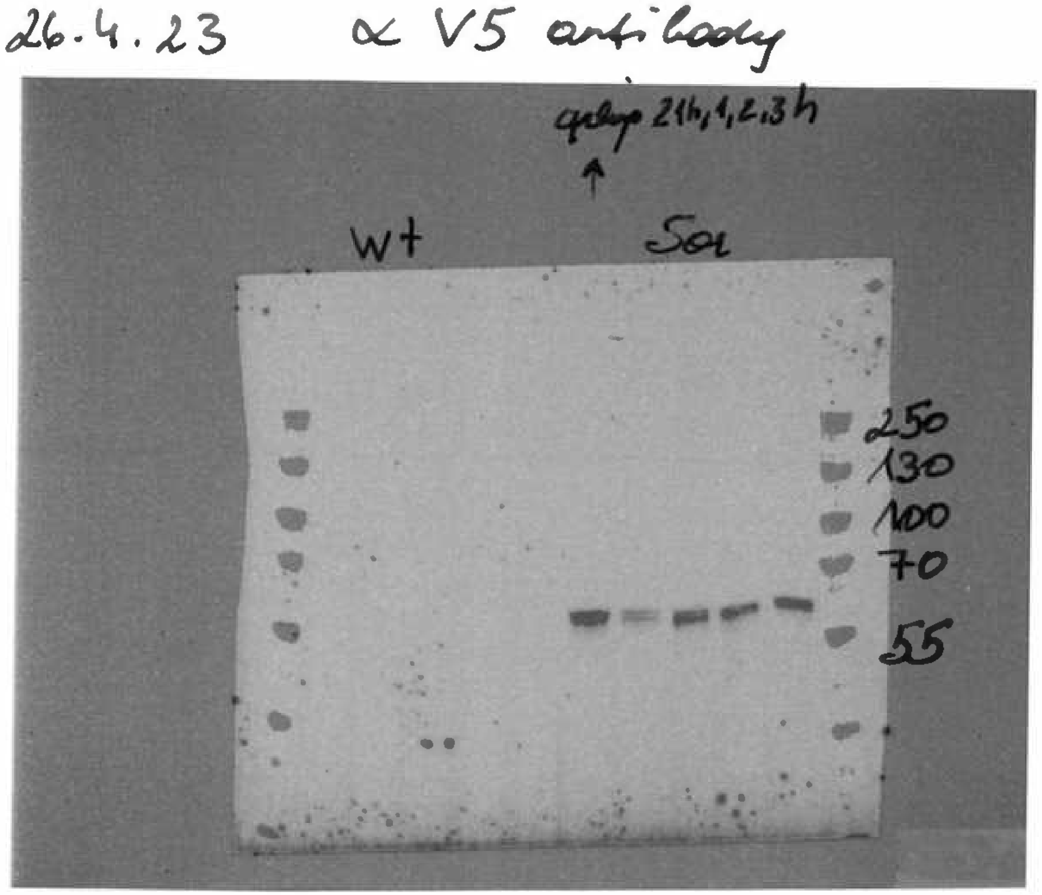

Sor1-pK

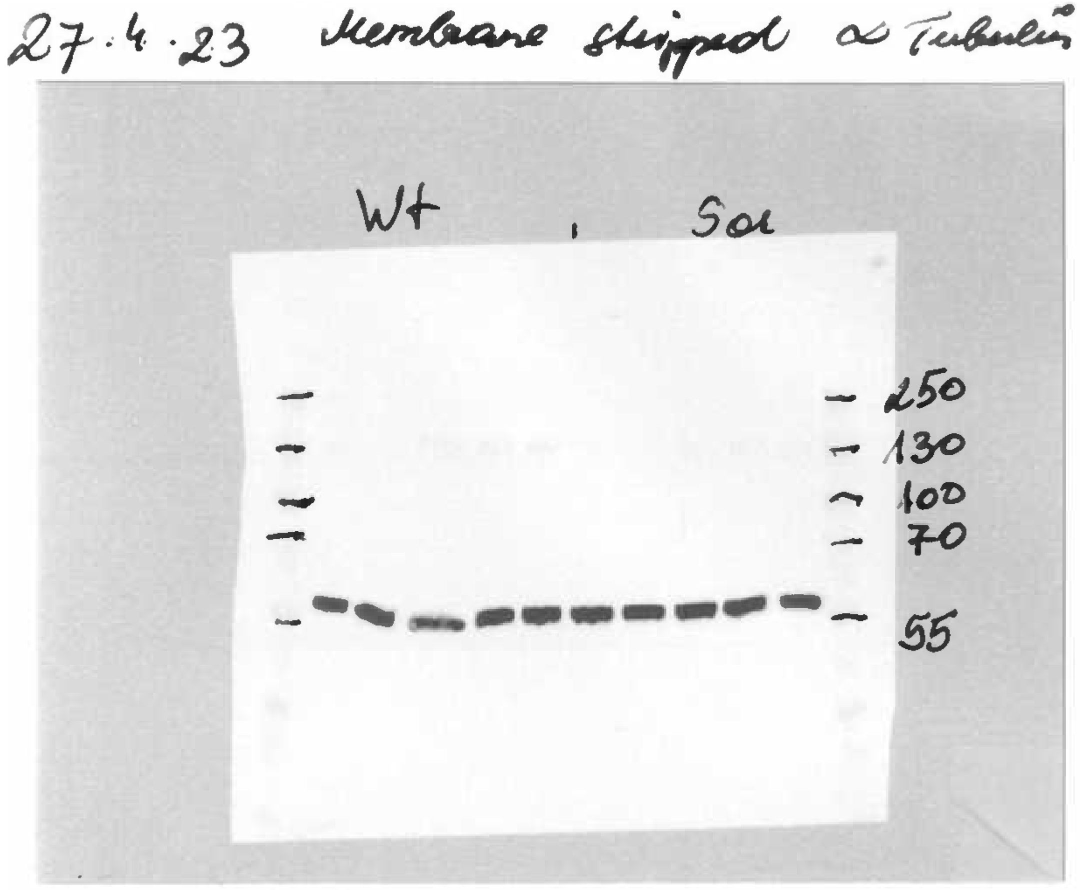

Tubulin

Supplementary Figure 2d

Interphase Sor1
and
endogenous
Sororin

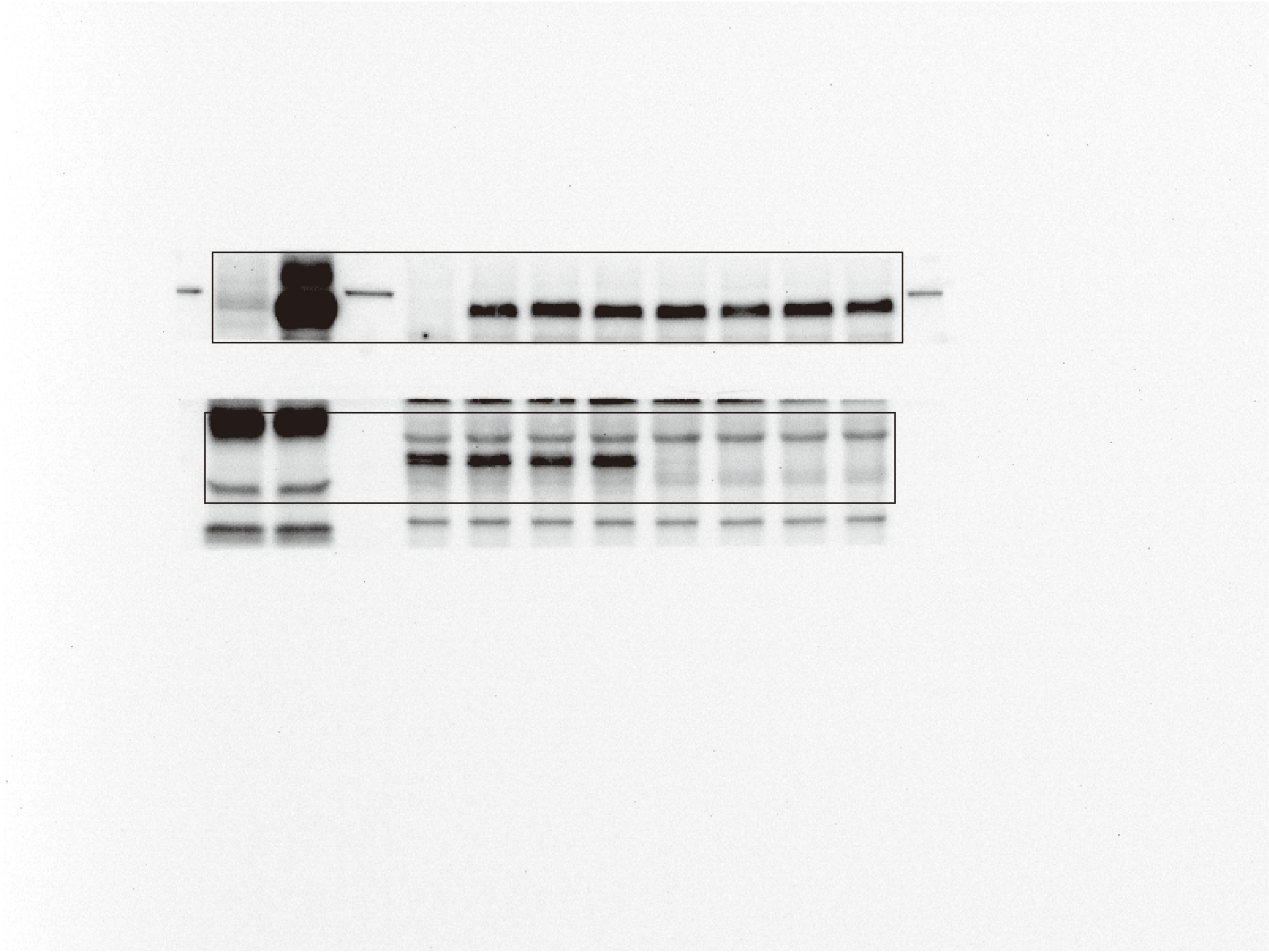

Supplementary Figure 2d

Mitosis Sor1

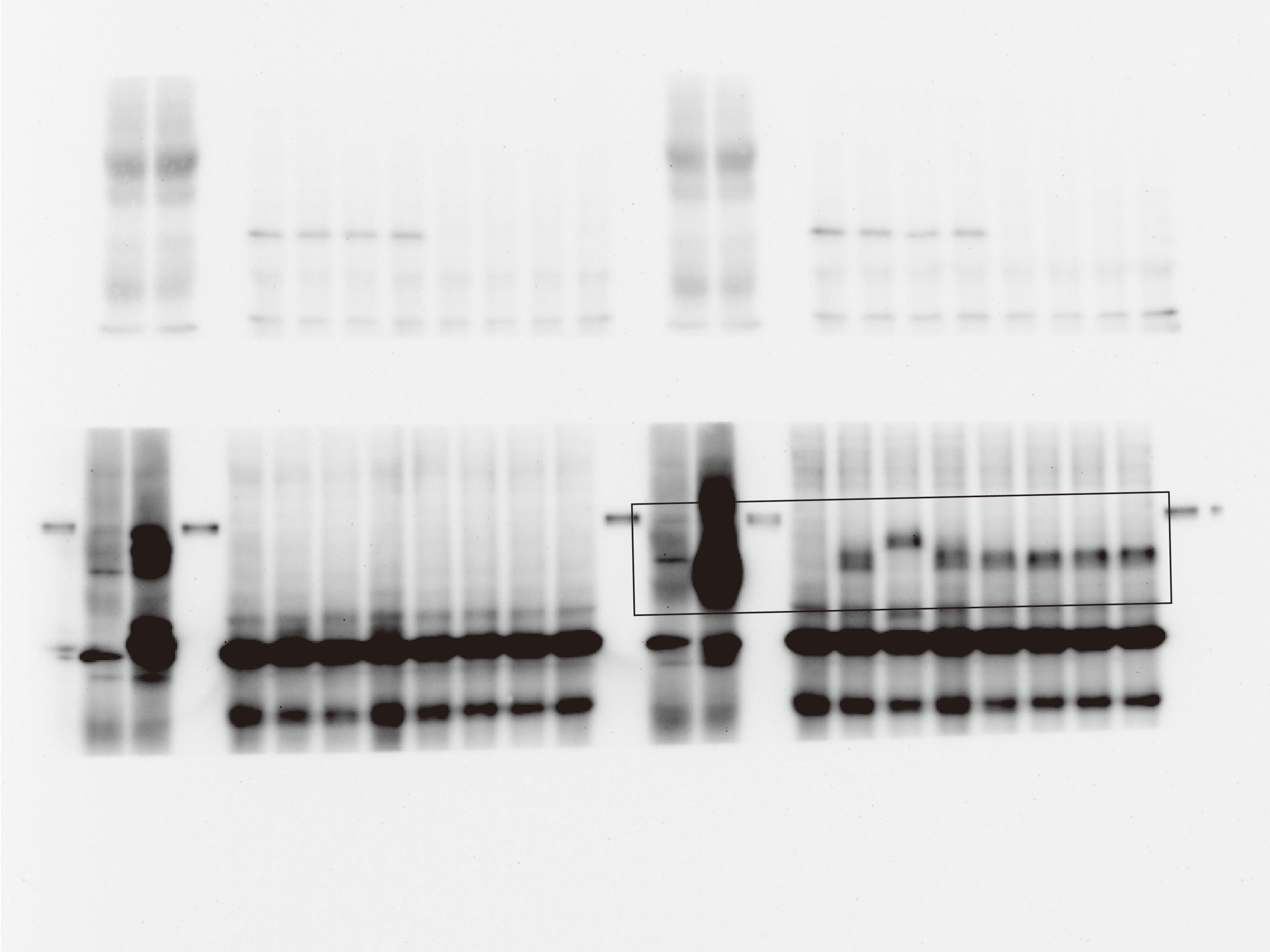

Supplementary Figure 2d

Mitosis cycB2

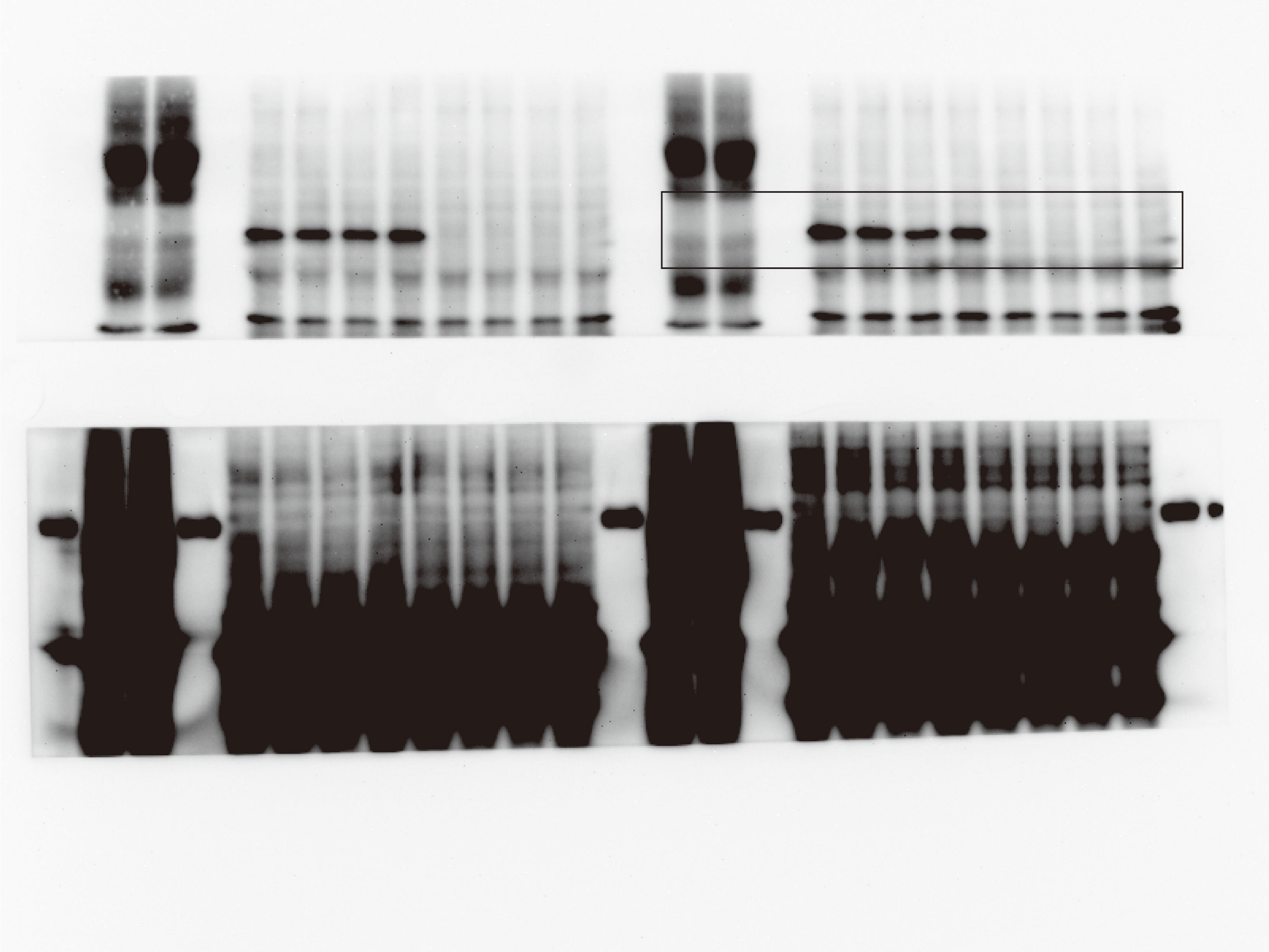

Supplementary Figure 2d

Ponceau

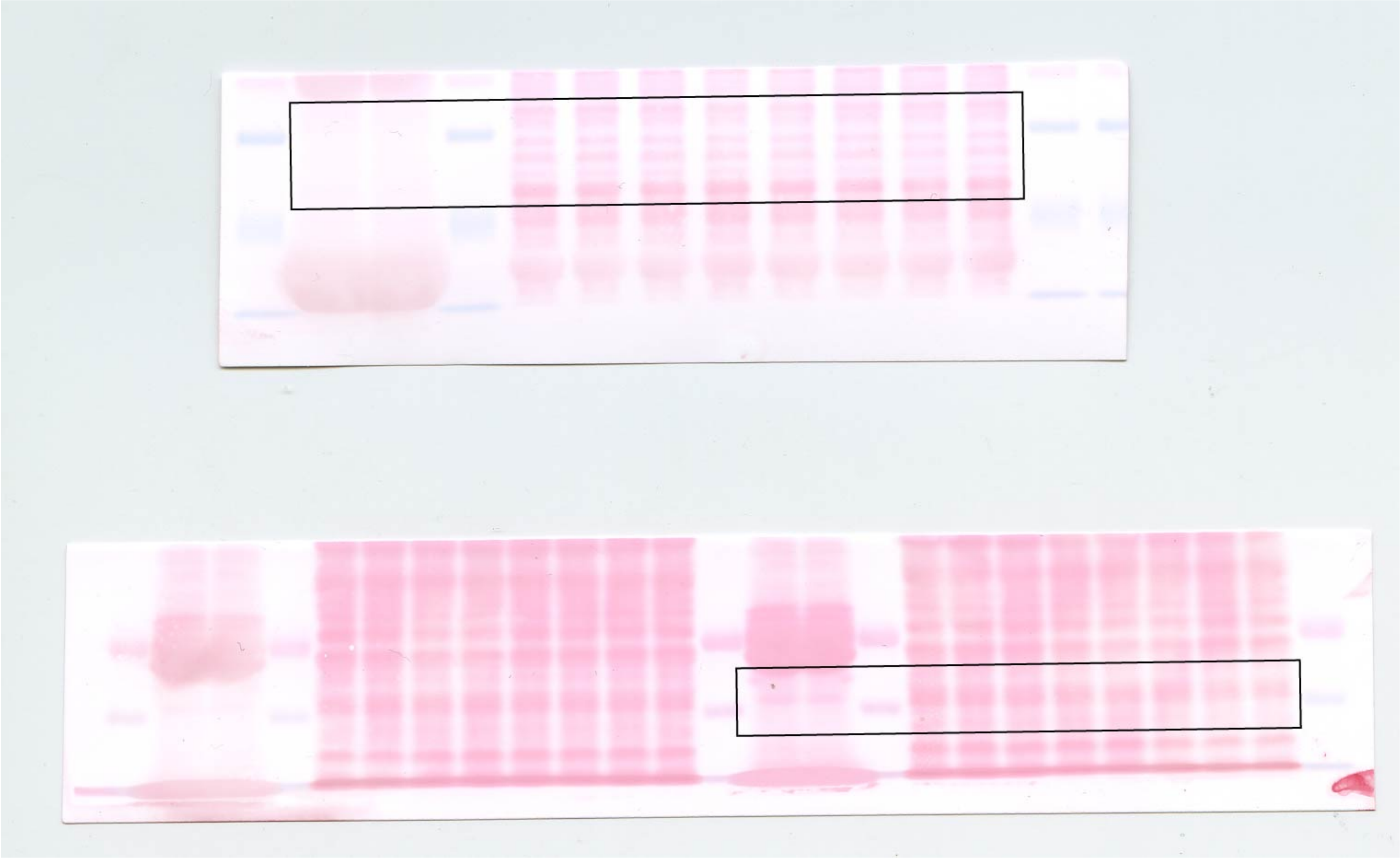

Supplementary Figure 2e

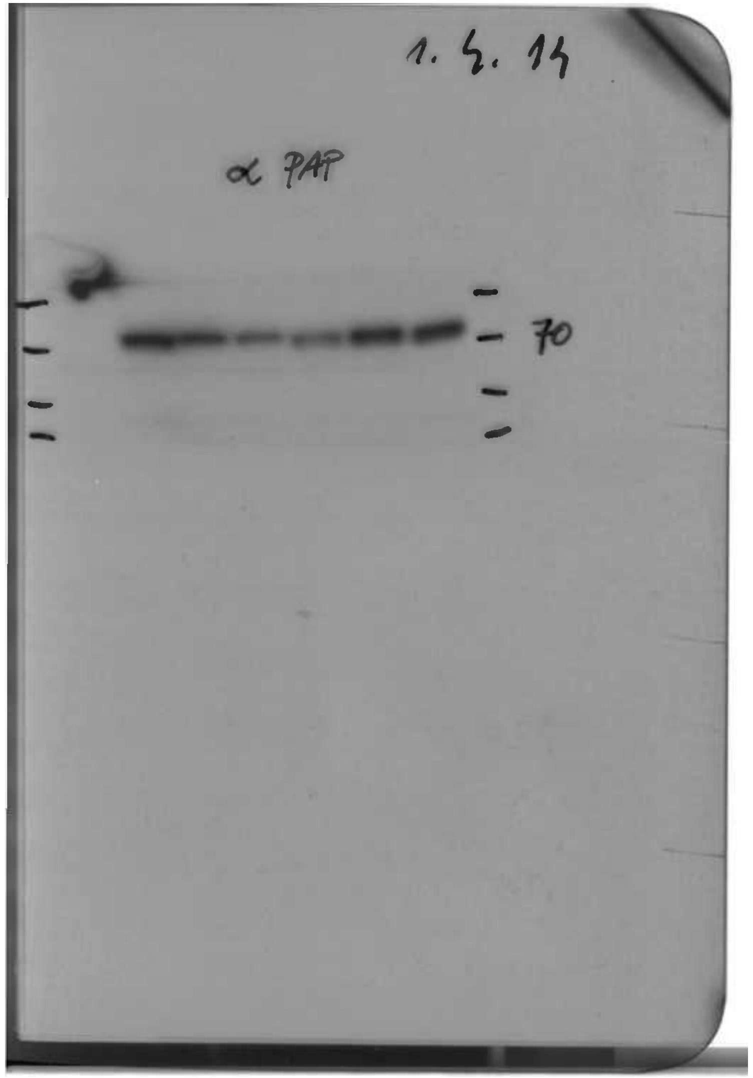

TAP

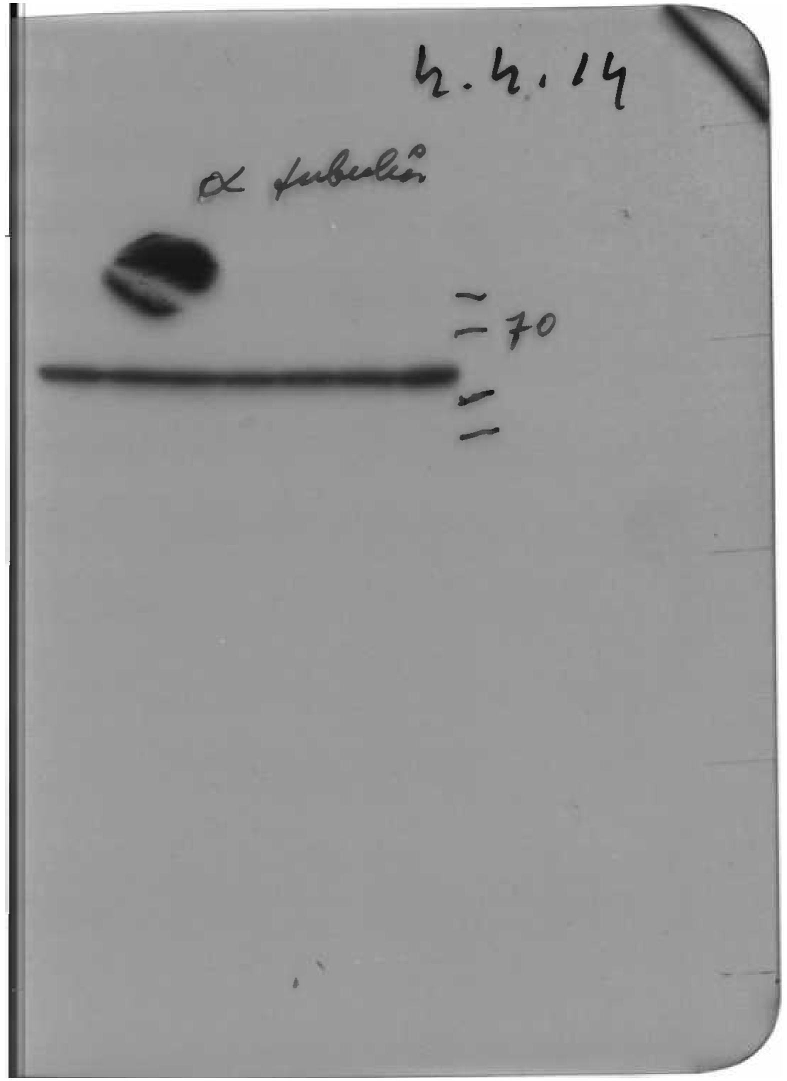

Tubulin
